# Comparative assessment of two commonly used commercial ELISA tests for the serological diagnosis of contagious agalactia of small ruminants caused by *Mycoplasma agalactiae*

**DOI:** 10.1186/1746-6148-8-109

**Published:** 2012-07-09

**Authors:** François Poumarat, Dominique Le Grand, Patrice Gaurivaud, Emilie Gay, Myriam Chazel, Yvette Game, Dominique Bergonier

**Affiliations:** 1 Anses, Lyon Laboratory, UMR «Mycoplasmoses of Ruminants», 31 Avenue Tony Garnier, F-69364, Lyon cedex 07, France; 2 UMR «Mycoplasmoses of Ruminants», Université Lyon1_F-69003, VetAgro Sup-Campus Vétérinaire de Lyon, F-69280, Marcy-L’étoile, France; 3 Laboratoire Départemental d’Analyses Vétérinaires, 321 chemin des Moulins, F-73024, Chambéry cedex, France; 4 Université de Toulouse, ENVT, UMR 1225 Interactions Hôtes - Agents Pathogènes, F-31076, Toulouse, France

## Abstract

**Background:**

Contagious agalactia (CA) of sheep and goats caused by *Mycoplasma agalactiae* is a widely occurring economically important disease that is difficult to control. The ELISA is commonly used for the serological detection of CA but it has some limitations and the performance of the available tests have not been properly evaluated.

Two commercial ELISA kits are widely used*,* one involving a fusion protein as target antigen and the other a total antigen. The objectives were to compare these tests by evaluating:

i. Their diagnostic sensitivity and specificity, the relevance of the recommended cut-off points, the correlation between the two tests, and, the correlation between serology data and the milk shedding of *M. agalatiae*;

ii. The influence of extrinsic factors such as the targeted animal species, geographical origin of the samples, intra-specific variability of *M. agalactiae* and concurrent mycoplasma infections.

A sample of 5900 animals from 211 farms with continuous CA monitoring for 20 years and no prior vaccination history was used. The infection status was known from prior bacteriological, epidemiological and serological monitoring with a complementary immunoblotting test.

**Results:**

The average diagnostic sensitivity was 56% [51.8–59.8] for the fusion protein ELISA and 84% [81.3–87.2] for the total antigen ELISA, with noteworthy flock-related variations. The average diagnostic specificity for the fusion protein ELISA was 100% [99.9–100], and for the total antigen ELISA differed significantly between goats and sheep: 99.3% [97.4–99.9] and 95.7% [93.8–97.2] respectively.

Experimental inoculations with different *M. agalactiae* strains revealed that the ELISA kits poorly detected the antibody response to certain strains. Furthermore, test performances varied according to the host species or geographical origin of the samples.

Finally, the correlation between milk shedding of *M. agalactiae* and the presence of detectable antibodies in the blood was poor.

**Conclusions:**

These serological tests are not interchangeable. The choice of a test will depend on the objectives (early detection of infection or disease control program), on the prevalence of infection and the control protocol used. Given the variety of factors that may influence performance, a preliminary assessment of the test in a given situation is recommended prior to widespread use.

## Background

Contagious agalactia (CA) is a disease of sheep and goats mainly characterized by mastitis with a subsequent drop in milk production. Mastitis is often associated with arthritis and/or kerato-conjunctivitis and sometimes with pneumonia and septicemia in young animals. Different species of mycoplasma (bacteria lacking a cell wall, in the class Mollicutes) cause CA. The main species in both sheep and goats is *Mycoplasma agalactiae* and three other species produce a clinically similar disease in goats: *M. capricolum* subsp. *capricolum, M. putrefaciens* and mainly *M. mycoides* subsp. *capri*[1]. *M. agalactiae* CA is found worldwide and is common in high milk-producing regions including countries bordering the Mediterranean sea [2]. The welfare and especially economic consequences of this disease justify its inclusion in the list of animal diseases of global concern established by the World Organization for Animal Health (OIE) (http://www.OIE.int).

Until recently, *M. agalactiae* CA was enzootic in two French regions: the northern Alps in goats (Savoie and Haute-Savoie departments) and the western Pyrénées (Pyrénées-Atlantiques (P.A.) department) in dairy sheep [3]. Mandatory screening based on serological ELISA tests was introduced into these areas 20 years ago. The disease appears to have been eradicated in the Alps as no *M. agalactiae*-infected herd has been identified for the last 10 years. In the P.A., *M. agalactiae* CA regularly regressed from 1993 to 2005 but then re-emerged abruptly. The number of outbreaks increased from 0 to 200 between 2006 and 2010, underlining the inadequacy or misapplication of the applied control methods [3].

Two methods are generally used for detecting *M. agalactiae-*infected farms (with or without clinical expression): indirect serological detection when animals are not vaccinated and direct evidence of the organism in milk (individual or bulk tank milks) [4]. The two methods are complementary: a direct search in tank milk is useful for early detection of newly infected herds while serological testing can be used on non-milk producers or to detect animals with chronic or latent infection where little or no mycoplasma are shed in the milk [2].

Several serological ELISA tests for *M. agalactiae* have been described and compared (Table 1) [4-8]. They use as target antigen, either the total antigens of *M. agalactiae* strain(s), or fusion proteins, copies of *M. agalactiae* specific immunogenic proteins. According to the literature, the diagnostic specificity (percentage of uninfected animals that test negative) is between 76 and 99% for tests using total antigens and between 97 and 100% for tests using fusion protein(s). The diagnostic sensitivity (percentage of infected animals that test positive) varies widely between tests (48 to 94%) and also according to the study for a given test (56 to 82%) [6-8]. Such differences may be partly explained by inadequate sampling, the different geographical locations of the studies (New Zealand, Brazil, Italy, France), the animal species targeted (goats or sheep) or the infection stage.

**Table 1:** **Literature data regarding the diagnostic specificity and sensitivity of ELISA tests used to detect antibodies against*M. agalactiae***

**Kit origin**	**Antigen type**	**Diagnostic specificity**	**Diagnostic sensitivity**	**Tested on**	**References**
Anses Sophia Antipolis France (a)	Total antigen 12 strains mixture	94%	48%	1017 sheep sera in 52 flocks from the French Pyrénées-Atlantiques department	[8]
Intervet(CHEKit®)(a)	Total antigen PG2 reference strain	99%	72%	1017 sheep sera in 52 flocks from the French Pyrénées-Atlantiques department	[8]
		99%	76%	30 goat sera collected from an infected herd and 97 uninfected sheep sera from New Zealand	[7]
		76%	74%	223 CA infected and 120 CA free sheep from Italy	[6]
POURQUIER-ELISA*M. agalactiae* (b)	Fusion protein P48	99%	82%	1017 sheep sera in 52 flocks from the French Pyrénées-Atlantiques department	[8]
		100%	56%	30 goat sera collected from an infected herd and 97 uninfected sheep sera from New Zealand	[7]
		-	57%	223 CA infected and 120 CA free sheep from Italy	[6]
Italy (c)	Fusion proteins P80 and P55	97%	94%	223 CA infected and 120 CA free sheep from Italy	[6]
Brazil (c)	Total antigen Unknown strain	95%	77*–*89%	86 sera from 44 bacteriologically positive and 42 bacteriologically negative Brazilian goats	[5]

(*a*) Formerly used commercial kit, (*b*) Test currently marketed and included in the study.

(*c*) Non commercial tests.

*CA*: contagious agalactia.

The objective of this study was to compare two ELISA tests that are widely used in Europe for the serological detection of *M. agalactiae* antibodies by evaluating: i) the performances *per se* : namely diagnostic sensitivity and specificity, the relevance of the recommended cut-off points, the correlation between the tests, and, the correlation between the presence of detectable antibodies and *M. agalactiae* shedding in milk; ii) the influence of extrinsic factors such as targeted animal species, geographical origin of samples, the intra-specific variability of *M. agalactiae* and mycoplasma co-infections.

## Methods

### ELISA kits

#### Description of ELISA kits

The « POURQUIER-ELISA *M. agalactiae* » kit (IDEXX-Institut Pourquier, 326 rue de la Galera, F-36097 Montpellier cedex 5, France) is an indirect ELISA test. It targets antibodies against a fusion protein equivalent to *M. agalactiae* P48 protein [9] (P48-ELISA kit) and uses an anti-immunoglobulin G (IgG) conjugate. Normalised values (NV) based on optical density (OD) are given by the following formula: NV = (sample OD - negative control OD) × 100/(average OD of the positive control - average OD of the negative control). Results are interpreted as follows: negative when NV ≤ 50%, doubtful when NV is between [50-60%] and positive when NV ≥ 60%.

The « LSIVET *M. agalactiae* » kit (Laboratoire Service International, 6 allée des Ecureuils Parc Tertiaire Bois-Dieu, F-69380 Lissieu, France) is an indirect ELISA test that targets antibodies against the total antigens (TA-ELISA kit) of a *M. agalactiae* strain isolated from a goat in Spain. It uses an anti-IgG conjugate. Normalised values (NV) are given by the following formula: NV = sample OD/(2 x negative control OD). Results are interpreted as follows: negative when NV < 0.8, doubtful when NV is between [0.8-1] and positive when NV > 1.

#### Measurement accuracy

The confidence interval for a single measurement was established from repeated measurements of a positive serum (PAL97) and three 2 by 2 dilutions of this serum on each test plate : 80 test plates of four different batches (20 plates by batch) for TA-ELISA kits and 60 test plates of two different batches (30 plates by batch) for P48-ELISA kits. The confidence interval on a single measurement was defined as +/− twice the standard deviation of the mean of the replicates [10]. PAL97 serum was used as the positive reference serum for an European serological inter-laboratory testing (http://www.OIE.int). It originated from an outbreak of *M. agalactiae* CA in P.A. and was obtained from a ewe eight months after clinical recovery.

### *M. Agalactiae* detection in milk

#### Methods

*M. agalactiae* detection in milk was performed in two steps. First, samples were enriched with mycoplasmas by two successive incubations at 37°C and 5%C0_2_ for four days into appropriate broth media (Oxoid), secondly the presence of *M. agalactiae* was fast screened by a sensitive real time PCR test that targeted a *M. agalactiae*/*M. bovis* specific sequence located on the gene coding for 16 S RNA [11]. Finally all cultures found positive or doubtful were tested again by a different PCR test that targeted the *M. agalactiae* specific P30 protein [12]. Only positive samples with both PCR tests were considered as positive in the study.

#### Assessment of *M. agalactiae* identification specificity

Three hundred cultures that were positive with the above-described method were also tested by a dot-immunobinding technique [13].

### Experimental infection of goats with different strains of *M. agalactiae*

Experimental infections were conducted with three different *M. agalactiae* strains: the PG2 reference strain, the 5632 strain isolated in Spain from a goat joint prior to 1991 [14] and the 14628 strain isolated in France from bronchopneumonia lesions in a wild *Capra ibex* in 2006 [3]. The Lyon Veterinary School Ethical Committee gave approval under agreement number #0730.

Test animals came from a goat herd that was certified free of pathogenic mycoplasma*.* Certification was based on regular bacteriological monitoring for the last five years (ear swabs, bulk tank and individual milk samples)*.* All three experiments were performed under identical conditions: 2 to 3 adult goats were inoculated subcutaneously with 2 ml of culture containing 10^8^–10^9^ CFU/ml twice at a 16-day interval for strains 5632 and 14628 or at a 23-day interval for strain PG2.

Blood samples were taken every 3 days after the first inoculation until slaughter, 20 to 35 days later. The serological response was monitored by immunoblotting test and with both P48- and TA- ELISA kits.

### Selection of infected reference animals and flocks

#### Selection of infected reference flocks

Six sheep flocks infected by *M. agalactiae* (Table 2) were selected from the P.A. department, where CA has re-emerged recently. The bacteriological, serological and clinical statuses of the P.A. flocks were well known after 20 years of continuous CA monitoring without any vaccination scheme. The infected reference flocks were approximately the same size, and contained only one breed of sheep (Manech) reared under similar animal husbandry conditions. In particular, breeding was synchronized, so that milk production started at approximately the same time for all sheep and lasted 8 to 9 months. However the anteriority of *M. agalactiae* infection in each flock ranged from one to eight years after the first *M. agalactiae* detection in bulk tank milk. The prevalence of mastitis, arthritis and kerato-conjonctivitis during the study period was low in all flocks, less than 6%, but acute CA clinical signs reappeared during the following two years in 5 out of the 6 flocks. No animal purchase or grouping with another flock (mountain pasture, winter pension) occurred during the study.

**Table 2:** **Prevalence of*M. agalactiae*infection and diagnostic sensitivity of TA- and P48-ELISA kits in six infected flocks**

**Flock characteristics**				**Sero-prevalence (a)**		**Diagnostic sensitivity (b)**			
**N°**	**Flock size (number of adults)**	**Number of infected animals (c)**	**First infection evidence n years ago (d)**	**TA-ELISA**	**P48-ELISA**	**TA-ELISA**		**P48-ELISA**	
				**% (e)**	**% (e)**	**% (g)**	**95% Confidence interval (f)**	**% (g)**	**95% Confidence interval (f)**
1	272	73	1	76 (206)	24 (64)	96 (70)	[88–99]	34 (25)	[24–46]
2	460	217	1	68 (312)	26 (119)	89 (193)	[84–93]	38 (82)	[31–45]
3	250	55	2	78 (194)	24 (59)	100 (55)	[94–100]	62 (34)	[48–75]
4	418	93	2	35 (147)	28 (119)	52 (48)	[41–62]	54 (50)	[43–64]
5	473	59	3	49 (230)	40 (188)	75 (44)	[62–85]	71 (42)	[58–82]
6	292	112	8	77 (226)	80 (234)	93 (14)	[86–97]	96 (107)	[90–99]
Total	**2165**	**609**		**61 (1315)**	**36 (783)**	**84 (514)**	**[81.3–87.2]**	**56 (340)**	**[51.8–59.8]**

(a) % of sero-positive animals in each flock.

(b) % of sero-positives in infected animals.

(c) Number of ewes that shed *M. agalactiae* (as determined by PCR testing of individual composite milk samples) at least once during four successive tests 5, 4, 3 and 1.5 months prior to blood sampling that took place 6 months after the beginning of lactation.

(d) Number of years between the present and first time *M. agalactiae* milk positivity was detected in the flock.

(e) Number of sero-positive animals in each flock.

(f) Exact binomial test distribution.

(g) Number of sero-positives in infected animals.

#### Selection of infected reference animals

Infected reference animals were chosen from the six infected reference flocks described above. These flocks were monitored regularly during the 2009 milking campaign. For each lactating animal: i) milk samples (composite milk) were collected monthly from the second month after weaning and tested for the presence of *M. agalactiae* and ii) one blood sample was taken after six months of milk production.

Animals shedding *M. agalactiae* in milk at least once in the samples taken 150, 120, 90 or 45 days before blood sampling were classified as infected.

### Selection of uninfected reference animals

Four separate and independent animal populations were used in this study: two goat, one sheep and one mixed sheep/goat populations (Table 3).

**Table 3:** Diagnostic specificity of TA- and P48-ELISA kits for five uninfected sheep or goat populations

						**TA-ELISA kit**		**P48-ELISA kit**	
**Animal species**	**Region**	**Control program**	***M. agalactiae*infection**	***M.m.capri*infection**	**Number of animals**	**Specificity %**	**95% confidence interval (a)**	**Specificity %**	**95% confidence interval (a)**
Goat	Poitou-Charentes	Non specific to CA	Very low prevalence	High prevalence	256	**98.2**	*[95.8***–***99.4]*	**100**	*[98.7–100.0]*
Goat	Haute-Savoie	Specific to *M. agalactiae*	Disease-free for the last 10 years	Unknown	1381	**99.7**	*[99.3***–***99.9]*	**100**	*[99.0–100.0]*
Goat	Béarn	Specific to *M. agalactiae*	Disease-free for the last 10 years	Unknown	275	**99.3**	*[97.4***–***99.9]*	**100**	*[98.7–100.0]*
Sheep	Béarn	Specific to *M. agalactiae*	Disease-free for the last 10 years	None (or very low)	604	**95.7**	*[93.8***–***97.2]*	**100**	*[99.4–100.0]*
Sheep	Tarn	None	CA never reported	None (or very low)	1195	**94.9**	*[93.5***–***96.1]*	**100**	*[99.7–100.0]*

(a) Exact binomial distribution.

M.m.capri = M. mycoides subsp.capri, CA: contagious agalactia.

#### Goats from the Poitou-Charentes region

Poitou-Charentes is the major area of goat milk production in France. The health status of the goat population is monitored regularly. Goats in this region are known to be frequently infected with *M. mycoides* subsp. *capri* but *M. agalactiae* is rare [15,16]. Sixteen herds that had experienced an episode of clinical CA caused by *M. mycoides* subsp. *capri* (confirmed after isolation in tank and individual milk samples) were selected: 10 in 2003 and 6 in 2007. Approximately 10 individual serum samples were collected from each herd selected in 2003 and 30 samples in 2007. A total of 276 goat sera were analyzed.

#### Goats from the Haute-Savoie department

The Haute-Savoie goat population has been under serological and bacteriological surveillance for *M. agalactiae* CA for over 15 years, and *M. agalactiae* infected herds have been systematically stamped out. No clinical outbreak of *M. agalactiae* CA has been detected since 2000. Sera collected during the 2007–2009 monitoring campaigns were used. 116 herds were selected at random: 87 contained fewer than 30 animals and 29 were large herds. All animals in the small herds were analyzed whereas only 20 randomly selected samples were analyzed in the large herds. In total, 1381 individual sera were analyzed.

#### Sheep from the Tarn department

The Tarn department is a major French producer of ewe’s milk with no history of CA. Sera from 21 flocks averaging 400 heads were collected in 2007 during a Maedi-Visna surveillance campaign. Sera from 50 to 60 adult animals from each flock were analyzed, providing a total of 1195 sera.

#### Mixed sheep and goat populations from Béarn

Only the western part of the P.A. department (Basque country) is infected with *M. agalactiae.* The eastern part (Béarn region) has been disease-free for 10 years following an eradication program with stamping out of *M. agalactiae* infected flocks. Samples were analyzed from: i) 604 sheep from 43 randomly selected flocks, at a rate of about 15 adults from each flock (average flock size 300 sheep) and ii) all 275 adult goats from 9 herds.

### Immunoblotting test (IBT)

#### Procedure

*M. agalactiae* PG2 strain clone 55–5 [14] was cultivated in liquid medium at 37°C for 48 hours. Cells were harvested by centrifugation at 12000 g for 20 min at 4°C followed by three washes in phosphate-buffered saline solution (PBS, 0.1 M Na_2_HPO_4_, 0.1 M Na_2_H_2_PO_4_, 0.15 M NaCl, pH 7.2). Washed cells were resuspended and stored at −20°C in the same buffer. The protein content of the cellular extract was determined as described by Lowry et al. [17]. Samples were mixed with lysis buffer (0.5 M Tris/HCl pH 6.8, 4.6% (w/v) SDS, 20% (v/v) glycerol, 10% (v/v) 2-mercaptoethanol and 0.004% bromophenol blue) and boiled for 5 min. Proteins were separated on a 4-12% gradient Bis-Tris polyacrylamide gel (Invitrogen). An electrophoresis was run on the Invitrogen Xcell surelock system following the manufacturer’s instructions. The separated proteins were transferred to a nitrocellulose membrane (Biorad) at 20 V constant voltage for 45 min in Tris glycine buffer (Tris 3.03 g/l, glycine 14.4 g/l) using the Biorad semi-dry transfer system. The membranes were blocked with a milk buffer (1 M glycine, 1% ovalbumin, 5% dry skim milk in PBS buffer) at room temperature and shaken for 2 hours then washed for 15 min at room temperature three times with PBS buffer 0.1% (v/v) tween 20 and finally with PBS alone. Each lane corresponding to a single well was cut, dried and immediately used or stored at −20°C. For immunoblotting, membranes were incubated for 2 hours at room temperature with 200 μl of serum diluted with 1 ml of dilution buffer (PBS containing 0.1% dry skim milk, 0.1% ovalbumin). After three washes in PBS 0.1% tween 20 and PBS buffer for 15 min at room temperature, the membranes were incubated for 2 hours at room temperature with an appropriate dilution of peroxidase-conjugated anti-sheep- or goat-IgG in dilution buffer then washed as described above. The peroxidase substrate containing 30 mg 4-chloro-1-naphthol dissolved in 10 ml methanol and 50 ml PBS and then 30 μl H_2_O_2_ was added and left in the dark for 5–15 min at room temperature.

#### Interpretation

A serum was considered positive if its profile contained four bands at 80, 48, 40 and 30 kDa simultaneously.

#### Validation

Three types of serum samples were used to validate the immunoblotting technique:

22 sera from infected reference ewes collected from the six infected reference flocks (Table 2). These animals shed *M. agalactiae* in milk and were positive with both P48 and TA-ELISA kits.

20 sera from ewes collected from flocks in disease-free areas and with no positive or doubtful ELISA result.

31 “doubtful” samples. These sera tested positive with the TA-ELISA kit. Animals originated from presumed *M. agalactiae*-free flocks that were thought to be free from *M. agalactiae*, based on bacteriological tank milk monitoring, but bordered the contaminated area of the Basque country.

### IBT re-assessment of ELISA positive results in presumed *M. Agalactiae* CA-free regions

When at least two animals from flocks located in CA-free areas tested positive with an ELISA test (2 to 12 per farm), the positive sera were re-assessed using IBT. A total of 20 sera from 5 flocks in the Béarn and 37 sera from 7 flocks in the Tarn were re-assessed.

### Statistical analysis

Data management was conducted with Excel. The correlation between results from different tests was calculated using the Kendall rank correlation coefficient (or Kendall’s tau coefficient) computed with R software (R Development Core Team, 2010, R Foundation for Statistical Computing, Vienna, Austria. http://www.R-project.org/). The other statistical analyses were conducted using the EpiInfo® software. Confidence intervals of proportions were calculated using an exact binomial distribution. Comparisons between proportions were based on chi-square tests with an alpha level of significance of 5%.

## Results

### Use of IBT to determine the infection status of animals with regard to *M. agalactiae*

**Figure 1:**
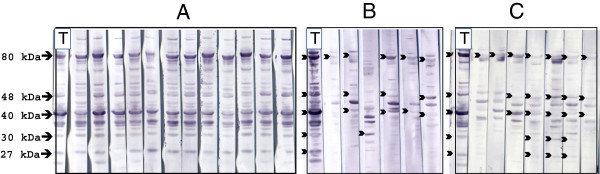
**Immunoblotting profiles of sheep sera collected in the Pyrénées-Atlantiques department.A**: sera from infected reference animals, **B**: sera from uninfected reference animals, **C**: sera from flocks thought to be uninfected but bordering newly infected flocks. Lane T: positive reference serum (PAL97). Black arrows indicate bands close to 80, 48, 40, 30 or 27 kDa. Total proteins from *M. agalactiae* PG2 reference strain clone 55.5 [14] were used as antigen.

**Figure 2:**
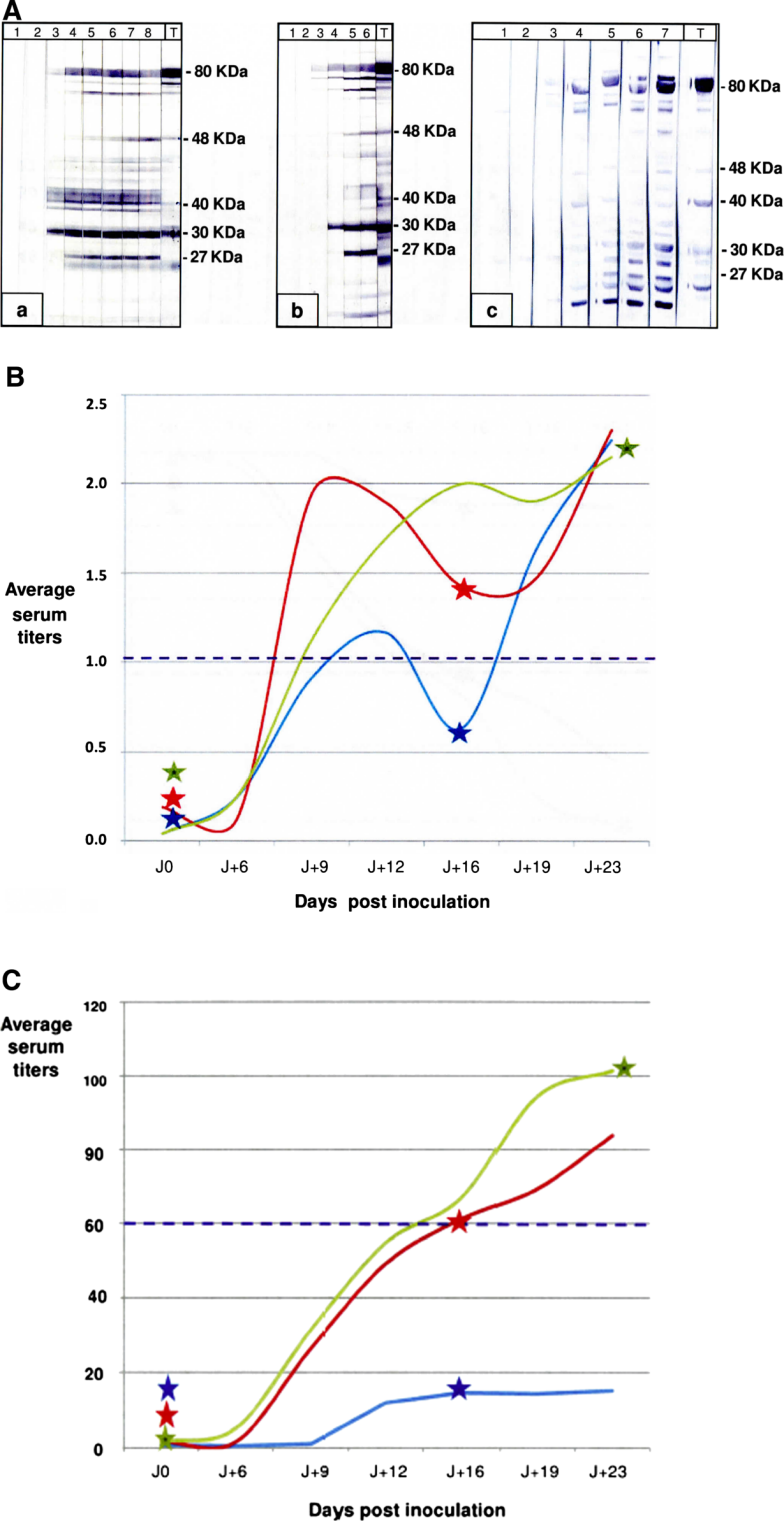
**Kinetics of serological responses in goats inoculated experimentally with three strains of*M. agalactiae.*** (**A**) Examples of immunoblotting obtained after inoculation: a) with PG2 reference strain; b) with 5632 strain; c) with14628 strain. Lane: 1 = J0, 2 = J + 6, 3 = J + 9, 4 = J + 12, 5 = J + 16, 6 = J + 19, 7 = J + 22, 8 = J + 30, days after first inoculation. Lane T: positive reference serum PAL97. Total proteins from the of *M. agalactiae* reference strain PG2 (clone 55–5) [14] were used as antigen. (**B** and **C**) Distribution of serum titers obtained respectively with TA-ELISA and with P48-ELISA. PG2: green curve, 5632: red curve, 14628: blue curve. Each curve corresponds to the average response of two animals for strains PG2 and 5632, three animals for strain 14628. Each point is an average of four repeated measures per animal. The manufacturer’s suggested cut-off point is represented by the purple broken line. * Days of inoculation are symbolized with a star. J + x: number of days after the initial inoculation.

All 22 sera from infected reference animals had an identical IBT profile characterized by simultaneous presence of four bands at 80, 48, 40 and 30 kDa and in most cases an additional band at 27 kDa (Figure 1-A). This profile was identical to the IBT profile obtained with sera from animals experimentally infected with the reference strain PG2 (Figure 2-A-a).

Several of the 20 sera from uninfected animals revealed IBT bands. However, the profiles were highly variable and never presented the 80-48-40-30 kDa bands simultaneously (Figure 1-B).

In general, the 31 doubtful samples had diverse profiles, all dissimilar to those of the infected reference animals. Only three samples (out of five) from one flock had profiles with bands at 80, 48, 40, 30 and 27 kDa (Figure 1-C). This farm experienced a clinical outbreak of CA in the following months.

### Correlation coefficient between the TA-ELISA and P48-ELISA kits

The apparent prevalence of infection in the population of 2165 lactating ewes was calculated from the six infected reference flocks in the P.A. (Table 2). On average, 61% of the animals were positive with the TA-ELISA kit and 36% with the P48-ELISA kit. However, these averages masked differences between flocks. Major differences between the two tests were apparent in the two flocks that had been recently infected (one year ago). The correlation coefficient (Kendall’s tau coefficient) between the two tests was 0.41 which is considered moderate.

### Diagnostic sensitivity of the TA-ELISA and P48-ELISA kits

Kit diagnostic sensitivity was estimated using sera collected from 609 infected reference ewes after six months of milk production (Table 2). The average diagnostic sensitivity of the TA-ELISA kit was 84% [81.3–87.2] and that of the P48-ELISA kit was 56% [51.8–59.8] (Table 2). The difference was significant (chi-square test, p < 10^-6^) indicating that the TA-ELISA kit was more sensitive. However, this average sensitivity masked major differences between flocks for both tests, ranging from 52% to 100% for the TA-ELISA kit and from 34 to 96% for the P48-ELISA kit. For the latter kit, the lowest values were obtained in recently infected herds.

### Diagnostic specificity of the TA-ELISA and P48-ELISA kits

Diagnostic specificity was estimated using 3711 sera from five distinct populations (Table 3). The specificity of the P48-ELISA kit was almost 100%, regardless of which *M. agalactiae* uninfected reference population was used. The specificity of the TA-ELISA kit varied from 95% to 100% according to the population and the animal species. Thus, 87 sheep in *M. agalactiae-*free populations (26 sheep from 14 flocks in the Béarn region and 61 sheep from 19 flocks in the Tarn department) gave positive results with the TA-ELISA kit. They were considered to be false positives as none of these sera had the IBT profile characteristic of *M. agalactiae* infection.

### Relevance of the manufacturer’s proposed cut-off points

#### Accuracy of a measurement

The confidence interval for a single measurement was approximately +/−20% regardless of the test, dilution of the reference serum or plate batch.

#### Distribution of titers according to proposed cut-off points and confidence interval on a single measurement

**Figure 3:**
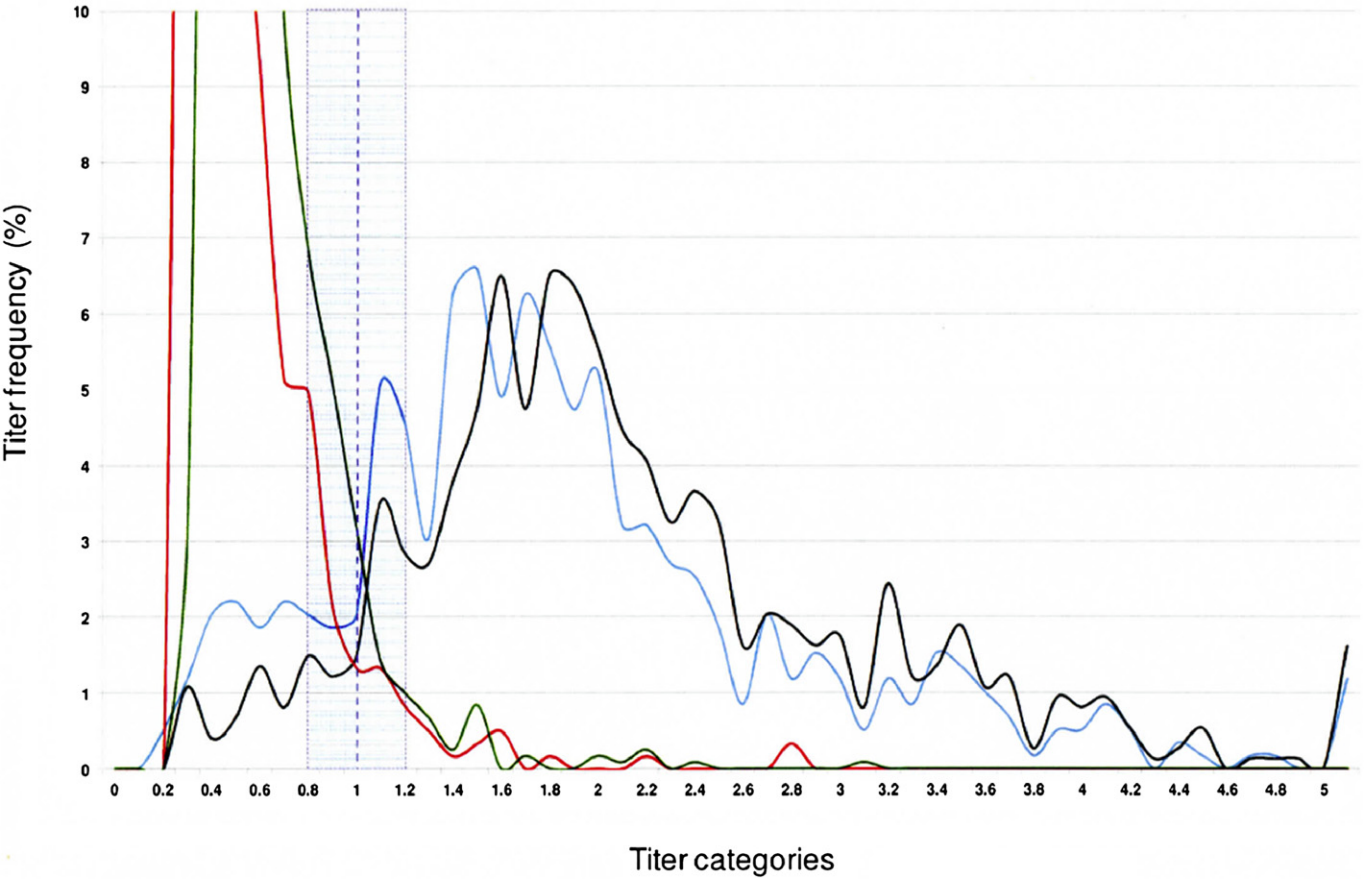
**Distribution of serum titers obtained with TA-ELISA in four distinct sheep populations: - green curve: 1195 sheep from the *M. agalactiae*infection-free population from the Tarn department; - red curve: 604 sheep from the*M. agalactiae*infection-free population from the Béarn region;- blue curve: 609 ewes that shed*M. agalactiae*in at least one out of four successive individual milk analyses 5, 4, 3 and 1.5 months prior to blood sampling for serological testing; - black curve: 783 ewes from six infected flocks from the Pyrénées-Atlantiques department that tested positive with P48-ELISA.** Titer distribution is expressed as the percentage of animals from a given population falling into in each titer category. The manufacturer’s suggested cut-off point is represented by the purple broken line. The shaded area corresponds to a +/−20% confidence interval on a single measurement. The maximum titer frequencies for the Tarn and Béarn populations were respectively at 0.5 (32% of the population) and at 0.4 (25% of the population).

**Figure 4:**
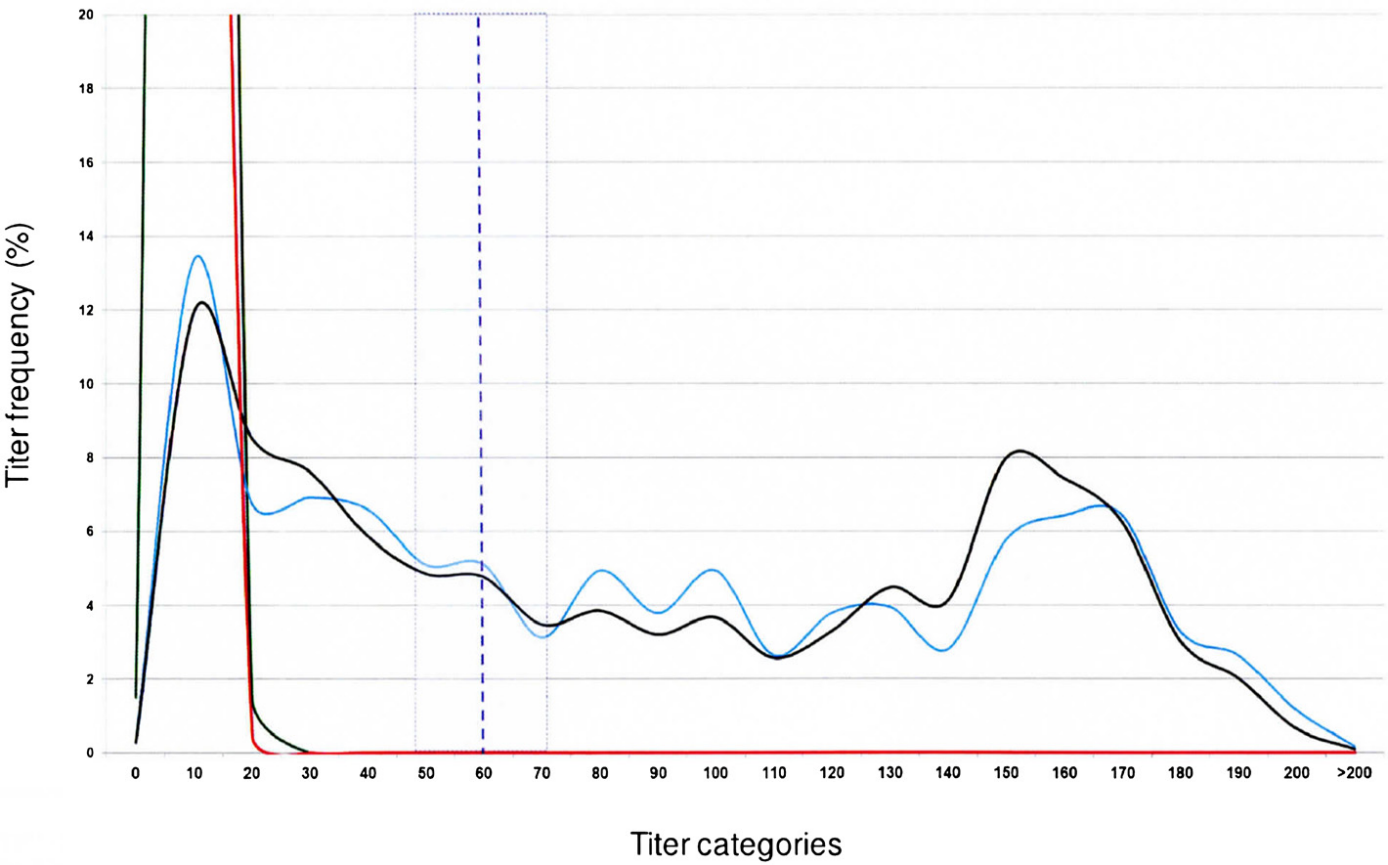
**Distribution of serum titers obtained with P48-ELISA in four distinct sheep populations: - green curve: 1195 sheep from the*M. agalactiae*infection-free population from the Tarn department;*-*red curve: 604 sheep from the*M. agalactiae*infection-free population from the Béarn region; - blue curve: 609 ewes that shed*M. agalactiae*at least one out of four successive individual milk analyses 5, 4, 3 and 1.5 months prior to blood sampling for serological testing; - black curve: 1090 animals from six infected flocks from the Pyrénées-Atlantiques department that tested positive with TA-ELISA.** Titer distribution is expressed as the percentage of animals from a given population falling into each titer category. The manufacturer’s suggested cut-off point is represented by the purple broken line. The shaded area corresponds to a +/−20% confidence interval on a single measurement. The maximum titer frequencies for the Tarn and Béarn populations were respectively at 10% (97% of the population) and at 10% (57% of the population).

For both the TA- and P48-ELISA kits, the distribution of serum titers in four distinct sheep populations was expressed as the percentage of animals from a given population which fell into each titer category (Figures 3 and 4). For each kit, the titer distributions for two “*M. agalactiae* infected” and two “*M. agalactiae* uninfected” populations were plotted on the same graph. These were the two uninfected sheep populations from the Tarn department and Béarn region, the 609 infected reference sheep and finally a “probably infected” population consisting respectively of: i) 783 positive animals with the P48-ELISA kit for the titer distribution of the TA-ELISA kit and ii) 1090 positive animals with the TA-ELISA kit for the titer distribution of the P48-ELISA kit.

The positive cut-off point suggested by the TA-ELISA kit manufacturer (Figure 3) showed a good fit with the intersection of the infected and uninfected population distributions (with a confidence interval for a single measurement about 20%).

The cut-off point of 60% suggested by the P48-ELISA kit manufacturer (Figure 4) appeared excessive, given the distribution of the titers in both uninfected populations where no titer exceeded 25%. For a given 100% specificity and 20% confidence interval, lowering the threshold to 50% or 40% or 35% would increase the sensitivity of the test from 56% to 61%, 66% and 70% respectively (with a confidence interval for a single measurement about 20%).

### Correlation between the serological results and excretion of the infectious agent

The correlation between the presence of detectable antibodies in the blood and *M. agalactiae* milk shedding was poor for both tests with a Kendall correlation coefficient of 0.31 for the TA-ELISA kit and 0.26 for the P48-ELISA kit. Respectively 31% and 15% of the ewes that were known to have shed *M. agalactiae* between 45 and 150 days prior to blood collection tested negative with the P48-ELISA and TA-ELISA kits. This result was not attributed to false positive PCR reactions. The *M. agalactiae* detection was specific, the first positive PCR were confirmed by a second independent PCR. In addition 299 (99.7%) of the 300 cultures that tested *M. agalactiae* positive with PCR were also *M. agalactiae* positive when further tested by dot-immunobinding.

### Factors that may affect the diagnostic performances of ELISA tests

#### Effects of *M. Agalactiae* intraspecific variability on ELISA test performances

The serological responses to experimental infections with three *M. agalactiae* strains (PG2, 5632 and 14628) were monitored by IBT, and with the TA-ELISA and P48-ELISA kits (Figure 2).

IBT showed that all goats developed a significant humoral immune response following inoculation regardless of the strain used. No band was identified by IBT on the inoculation day, and multiple bands appeared from the 16^th^ day after inoculation (Figure 2A).

Animals inoculated with strains 5632 and PG2 tested positive nine days later with the TA-ELISA kit and 16 days later with the P48-ELISA kit (Figures 2B and 2C). However the increase in antibodies of animals inoculated with strain 14628 was only weakly detected by the TA-ELISA kit and not at all with the P48-ELISA kit, despite a booster shot 16 days after the initial inoculation (Figures 2B and 2C).

#### Effects of the host species and geographic location on the ELISA kit performance

In this study, the specificity of the TA-ELISA kit appeared to be lower in sheep (on average 95%) than in goats (99%). Specificity differed significantly between sheep and goats from the same geographic region (Béarn) (p = 0.005).

The specificity of the TA-ELISA kit differed significantly between three goat populations from three different geographical locations: Haute-Savoie, Poitou-Charentes and Béarn (p = 0.008). However, the specificity did not differ significantly between the two sheep populations from the Tarn department and Béarn region (p = 0.45).

#### Effects of concurrent infection with other mycoplasma species that cause CA on ELISA test performances

In the 256 animals in the 16 herds currently infected with *M. mycoides* subsp. *capri,* only five in three herds were positive with the TA-ELISA kit, corresponding in 98% of specificity that was similar to that of other goat populations (Table 3).

## Discussion

This study compared two commercial ELISA kits widely used in Europe to detect antibodies against *M. agalactiae,* the primary agent of CA. Kits were tested on a large number of sera collected from 5900 animals from 211 farms. The constitution of representative samples of farms with well known infection status was possible for several reasons: first, the epidemiological situation of CA in France is well surveyed [3], second, a large number of farms and animals had been monitored for many years as part of several distinct eradication campaigns and third, animals had not been vaccinated against this agent. This large sample size guaranteed the statistical reliability and accuracy of the study. Nevertheless, the average performance values should be regarded with caution because a sampling bias may still have occurred, and furthermore, the values varied according to the region, host species or *M. agalactiae* strain.

Accurate determination of the infection status of individual animals regarding *M. agalactiae* is difficult in the absence of a gold standard diagnostic technique. Thus, the criteria used to define animals as “infected reference” differ from one study to another and may be clinical, bacteriological or serological [5-8]. The OIE recommends that in the absence of a gold standard for classifying an animal as infected, isolation of the disease agent can legitimately be considered the method of choice [10]. Classification of an animal as infected may be biased because animals may shed the organism early in the course of infection even if the humoral immune response is still low. Furthermore, animals with latent infection that have developed an immune response may intermittently or no longer shed the organism and are therefore difficult to detect.

Here, the “infected reference” status of an animal was based on bacteriological identification of *M. agalactiae* in individual milk samples. The *M. agalactiae* detection protocol used in this study is highly sensitive and specific. Sensitivity was provided by the first step of culture: an inter-laboratory assay including 32 French diagnosis laboratories demonstrated that 70% of them detected *M. agalactiae* by culture when test aliquots contained only 2 to 25 CFU and 100% when test aliquots contained 25 to 250 CFU [unpublished data]. Specificity was provided by using two sets of PCR primers for the identification of *M. agalactiae*, and was confirmed by dot-immunobinding. Finally, bias associated with intermittent shedding and with early infection were limited by i) repeating bacteriological examinations of individual milk samples at regular intervals and ii) not taking into account milk samples performed during the period of 45 days prior to blood sampling.

The uninfected reference animals were recruited from geographical areas where *M. agalactiae-*related CA had never been reported or where routine monitoring had been in place for over 20 years and where no case had occurred in the last 10 years. Different independent populations and animal species (goats, sheep) were included to ensure diversity. Nevertheless, a number of animals, sometimes from the same farm, gave positive results with the TA-ELISA kit at the threshold recommended by the manufacturer. These positive results could have resulted from insufficient test specificity or from individual infections that escaped prior detection. A further technique was needed to unequivocally determine the status of these animals.

IBT is used for the serological diagnosis of contagious bovine pleuropneumonia (CBPP), another mycoplasma disease of ruminants, when routine tests are insufficiently accurate or ambiguous [18]. Sera from infected animals give five specific IBT antigenic bands at 110, 98, 95, 60 and 48 kDa simultaneously (http://www.oie.int). IBT has been used in several studies to identify *M. agalactiae* infection in cases of CA [6,7,19,20]. These authors described five stable immunogenic proteins with specific molecular masses of 80, 55, 48, 40, and 30 kDa, which presumably could be used as a specific signature of *M. agalactiae* infection much like with CBPP. However, three proteins (55, 30 and 40 kDa) are not expressed by all strains [6,12,21]. Thus an *M. agalactiae*-IBT should only be interpreted in the light of the antigenic profile of local strains.

A thorough analysis of 63 isolates collected between 1977 and 2007 in the P.A. showed that a single strain, very similar to the reference strain PG2, had been present for 30 years in the P.A [22]. This strain expressed the 80, 48, 40, and 30 kDa proteins but not the 55 kDa one identified in Sardinian strains [19]. A posteriori, the 55 kDa protein appeared to be antigenically similar to a variable protein of *M. agalactiae* known as Vpma U [6]*.* On the PG2 antigenical phenotype used in IBT, Vpma U is apparent at 27 kDa. Understandably, if a single *M. agalactiae* strain, similar to PG2 reference strain, was circulating in the P.A., it seems reasonable to suspect that all serum samples from infected animals would express the same IBT profile with four major bands corresponding to stable proteins and a 27 kDa band, as confirmed by analysis of the 22 sera from infected reference animals. In contrast, the profiles of uninfected animals were heterogeneous and bands at 80, 48, 40, 30 kDa never occurred simultaneously. One recently infected flock that had escaped monitoring was detected by IBT. So IBT proved to be useful in determining the status of P.A. flocks with regard to *M. agalactiae* when the ELISA test results were ambiguous.

Several sera from the uninfected population gave positive results with the TA-ELISA kit. Even in the most doubtful cases (several positives in one farm), the IBT profiles were never similar to those of infected animals. Presumably, these were false positives and the wide distribution of non-negative responses to the TA-ELISA kit on several farms would support this hypothesis.

Variability within *M. agalactiae* strains can affect test performances, and test sensitivity may be improved by using a local strain as antigen [23]. In the present study, the kinetics of antibody response were followed after experimental inoculations of goats with three different *M. agalactiae* strains. Detection of the immune response to one strain was poor with both ELISA kits. The variability of *M. agalactiae* is expressed in two ways. First, not all strains express certain specific immunogenic stable proteins [12-21]. Second, the expression and size of several surface proteins, such as the Vpma family, can show rapid and random fluctuations, thereby generating multiple antigenic surface configurations and broad intra-clonal variability [24]. These two concomitant phenomena may alter the host’s immune response, thereby affecting the sensitivity of diagnostic tests based on the degree of proximity between the phenotype of the test antigen and the phenotypes of the infecting strain. The consequences of antigenic variability on test performance are difficult to predict from strain characteristics. For example, both ELISA kits detect an immune response to strain 5632 even though this strain is genetically very different from the reference strain PG2 [14].

Test performance is also modified by geographic region and host species (sheep or goats). Regional differences may be due to antigenic variations between locally circulating strains. The TA-ELISA kit is less specific when used to detect *M. agalactiae* antibodies in sheep than in goats. This may be because it was developed with a goat strain that is possibly “adapted” to this species. Test specificity is not affected when goats are infected concurrently with other mycoplasma(s), even though these organisms may have many genes in common with *M. agalactiae*[25]. However, given the genetic and antigenic variability of *M. mycoides* subsp. *capri*[15,26], certain strains may well express atypical cross antigens, as is the case with CBPP [27].

The main source of CA contagion is by *M. agalactiae* shedding in milk [1,2]. This study showed that the correlation between milk shedding and a detectable serological IgG response in blood was poor, implying that serology should not be used as an indicator of infectiousness. The percentage of animals that shed and tested negative with the TA-ELISA kit varied from 0 to 48% (average 16%) between farms, and from 4 to 62% (average 31%) with the P48-ELISA kit. Reports in the literature concerning the duration of the humoral response to mammary infection show considerable divergence, ranging from a rapid, systematic and persistent sero-conversion of at least 320 days [28] to a highly transient sero-conversion of less than 32 days [29]. Further studies with reliable tests will be required to see whether the negativity in some infected animals results from the absence of serological response or from the rapid decrease of antibodies.

## Conclusion

Two commercially available ELISA kits are widely used in several European countries to detect IgG antibodies against *M. agalactiae*.

The P48-ELISA kit is highly specific but not very sensitive. Its analytical performance characteristics may retard the detection of newly infected flocks especially when an insufficient number of animals are sampled. It is best used at the flock level.

The TA-ELISA kit is more sensitive, especially early in infection. On the other hand, it lacks specificity, especially with regard to sheep. When infection prevalence is low, false positive reactions hamper the monitoring and have to be tested again with expert serological test such as IBT. But no universal and standardized IBT test has been published for *M. agalactiae* yet.

In practice, the choice of kit should depend on: the user’s objective (early detection of infection or ongoing control program), the epidemiological situation (prevalence) and the type of disease control program. The present ELISA kits because they do not detect all animals that are shedding *M. agalactiae*, are not well appropriate to estimate the infectious risk of a herd.

The analytical performance of both ELISA kits is highly subject to variations depending on the characteristics of the infecting strains. A preliminary assessment of their performance in a new situation is recommended prior to widespread use.

Future serological tests should involve several fusion proteins which reproduce antigens that are complementary, specific, stable and widely representative of *M. agalactiae* strains and furthermore are able to differentiate between responses to natural and vaccine antibodies.

## Competing interests

The authors have declared no conflict of interest.

## Authors’ contributions

FP conceived, coordinated and drafted the manuscript, DB coordinated all epidemiological and bacteriological studies in the P.A. and provided sera from the Tarn, MC and EG performed the statistical analysis, PG developed the immunoblotting test, YG coordinated serological monitoring in Haute-Savoie, DLG was in charge of experimental infections. All authors helped to improve the manuscript and approved the final version.
